# Redox Potentials of Magnetite Suspensions under Reducing Conditions

**DOI:** 10.1021/acs.est.2c05196

**Published:** 2022-11-17

**Authors:** Thomas C. Robinson, Drew E. Latta, Johna Leddy, Michelle M. Scherer

**Affiliations:** † Department of Civil and Environmental Engineering, University of Iowa, Iowa City, Iowa52242, United States; ‡ Department of Chemistry, University of Iowa, Iowa City, Iowa52242, United States

**Keywords:** iron oxide, redox potential, electron transfer, contaminant reduction, maghemite, magnetite

## Abstract

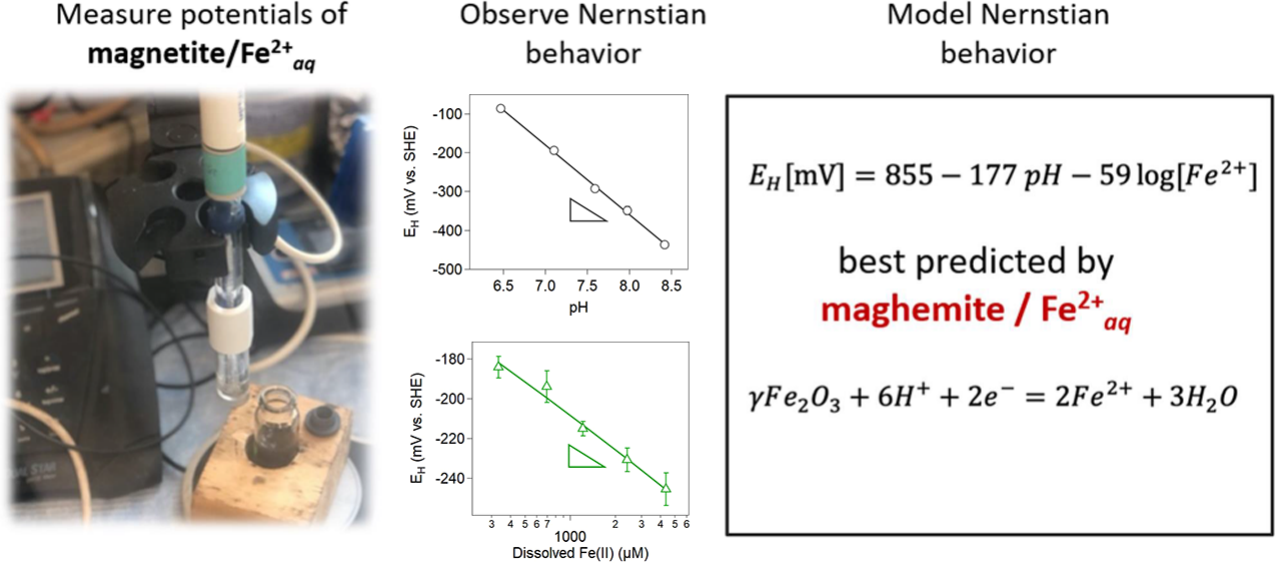

Predicting the redox behavior of magnetite in reducing soils and sediments is challenging because there is neither agreement among measured potentials nor consensus on which Fe(III) | Fe(II) equilibria are most relevant. Here, we measured open-circuit potentials of stoichiometric magnetite equilibrated over a range of solution conditions. Notably, electron transfer mediators were not necessary to reach equilibrium. For conditions where ferrous hydroxide precipitation was limited, Nernstian behavior was observed with an *E*_H_ vs pH slope of −179 ± 4 mV and an *E*_H_ vs Fe(II)_aq_ slope of −54 ± 4 mV. Our estimated *E*_H_^o^ of 857 ± 8 mV closely matches a maghemite|aqueous Fe(II) *E*_H_^o^ of 855 mV, suggesting that it plays a dominant role in poising the solution potential and that it’s theoretical Nernst equation of *E*_H_[mV] = 855 – 177 pH – 59 log [Fe^2+^] may be useful in predicting magnetite redox behavior under these conditions. At higher pH values and without added Fe(II), a distinct shift in potentials was observed, indicating that the dominant Fe(III)|Fe(II) couple(s) poising the potential changed. Our findings, coupled with previous Mössbauer spectroscopy and kinetic data, provide compelling evidence that the maghemite/Fe(II)_aq_ couple accurately predicts the redox behavior of stoichiometric magnetite suspensions in the presence of aqueous Fe(II) between pH values of 6.5 and 8.5.

## Abstract 2

Measured equilibrium potentials for magnetite−aqueous Fe(II) suspensions appear to be poised by the maghemite−Fe(II)_aq_ redox couple under circum-neutral, reducing conditions and may be useful for predicting contaminant reduction by magnetite.

## Introduction

Iron (Fe) oxides are ubiquitous in soils and sediments and play critical roles in contaminant remediation in the environment, biological respiration, and nutrient cycling.^1−11^ Fe oxides are redox active and cycle between ferrous and ferric Fe, releasing aqueous Fe(II) into the subsurface that substantially affects subsurface geochemistry.^10,12^ Magnetite, a mixed valent Fe(II)/Fe(III) mineral, forms in soils and sediments under reducing conditions and has been shown to reduce several contaminants.^3−5,13−25^ Magnetite’s ability to store and release charge during microbial respiration, contaminant remediation, and nutrient cycling has been highlighted as a potential biogeobattery in the environment.^10,26,27^ Because of its high conductivity, magnetite also has broad uses in industrial and technological materials and processes, such as nanofuels, capacitors, and batteries.^3,28−30^ Magnetite redox properties have been widely explored for these applications, as well as their role in natural and engineered environments relevant to contaminant behavior.^14,17,28,31−34^

Several methods have been used to measure or estimate magnetite redox potentials, including combination platinum redox probes,^17,32,35,36^ modified and unmodified rotating disc electrodes,^15,16,28,34,37^ and colorimetric absorbance measurements.^38^ Multiple methods, as well as experimental conditions, have resulted in a wide range of potentials reported for magnetite (from +560 to ∼−500 mV vs SHE over a pH range of 2 to 12^15−17,33,34,39,40^). In addition to the lack of agreement among reported potentials, estimating the potential of magnetite in soils and sediments is further complicated by magnetite being a mixed-valence mineral which can contain Fe^2+^ ions in the structure and result in a range of magnetite stoichiometries (*x* = Fe(II)/Fe(III)) which has been modeled as a magnetite–maghemite solid solution upon dissolution or oxidation.^17,32,36,41^

The most common equilibria used to interpret magnetite redox potentials are magnetite|Fe(II)_aq_, maghemite|magnetite, and hematite|magnetite (eqs 1, 2, and 3).^15,17,32,42^ Even among these, the reported standard redox potential (*E*_H_^o^) estimated from thermodynamic data can vary by up to 210 mV vs SHE. In addition to these three redox couples, lepidocrocite|Fe(II)_aq_, maghemite|Fe(II)_aq_, and lepidocrocite|magnetite (eqs 4, 5, and 6) have also been suggested as poising potentials in magnetite suspensions based on Mössbauer spectroscopy results and a linear free energy relationship (LFER) developed for nitroaromatic reduction rates and Fe oxide redox potentials.^23,32,38^ Where relevant, the range of calculated redox potentials reported is shown next to each redox couple in eqs 1–6. The variation in potentials is because different thermodynamic databases, and more importantly slightly different thermodynamic values, were used in different studies (Table S1). While most of the databases provide reasonably consistent free energies, there remains some variation in calculated redox potentials based on the database used.^43^ Both the lack of agreement among measured potentials and the ambiguity regarding which conceptual model and which Fe(III)|Fe(II) equilibrium reactions are most relevant make it challenging to predict magnetite redox behavior in soils and sediments.^15,17,32^

Magnetite|Fe(II)_aq_^15,17,32^
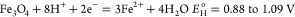
1

Maghemite|Magnetite^15^
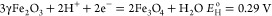
2

Hematite|Magnetite^15,17,42^
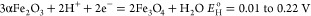
3

Lepidocrocite|Fe(II)_aq_^15^
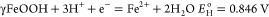
4

Maghemite|Fe(II)_aq_
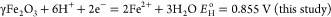
5

Lepidocrocite|Magnetite
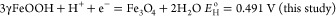
6

The objective of this study is to improve our ability to predict the behavior of magnetite in reducing soils and sediments by evaluating which redox couple poises the redox potential in magnetite suspensions under reducing conditions. To achieve this objective, we measured open-circuit potentials of stoichiometric magnetite equilibrated over a range of pH and Fe(II)_aq_ concentrations. We collected our data with one method to produce a consistent set of experimental conditions for us to evaluate our results. We used stoichiometric magnetite because under reducing conditions where aqueous Fe(II) is likely to be present, it has been shown that nonstoichiometric magnetite is recharged by oxidative sorption of aqueous Fe(II).^4,36,44^ Our findings indicate that the redox potentials of magnetite and Fe(II)_aq_ agree most closely with the maghemite|Fe(II)_aq_ redox couple. Our findings suggest that there are conditions where the complex chemistry of magnetite under reducing conditions can be well-described by a single redox reaction and, importantly, that a model that relies on pH, aqueous Fe(II), and redox potentials of relevant reactions may be achievable.

## Materials and Methods

### Mineral Synthesis and Characterization

The minerals used here were synthesized with previously reported methods adapted from Cornell and Schwertmann.^4,6,21,45^ Mineral purity was confirmed using powder X-ray diffraction (XRD) (Rigaku MiniFlex II, Co K_α_ radiation). Goethite was prepared from 100 mL of 1 M ferric nitrate (Fe(NO_3_)_3_·9H_2_O) solution by adding 180 mL of 5 M KOH and diluting to 2 L with 1720 mL of deionized (DI) water. The resulting suspension was placed in an oven at 70 °C for 60 h. The goethite was washed, centrifuged, freeze-dried, ground with a mortar and pestle, and sieved using a 100-mesh sieve (150 μm). Stoichiometric magnetite was prepared under anoxic conditions in a glovebox (93% N_2_/7% H_2_) from iron chloride salts (0.1 M FeCl_2_·4H_2_O and 0.2 M FeCl_3_·6H_2_O) added in a 1:2 Fe^2+^/Fe^3+^ ratio. The salts were dissolved in DI water and titrated with 10 M NaOH to above pH 10.0 to precipitate magnetite overnight in 2 L polypropylene containers. The mineral suspension was vacuum filtered and then freeze-dried. The freeze-dried particles were returned to the glovebox, ground in a mortar and pestle, and then sieved through a 100-mesh sieve (150 μm) (BET specific surface areas = 58 m^2^/g which is comparable to other magnetites synthesized in our group).^34^ Magnetite stoichiometry was measured by dissolving the synthesized magnetite in 5 M HCl where Fe(II) content was quantified with the 1,10-phenanthroline method. Magnetite stoichiometry was also assessed with XRD and Mössbauer spectroscopy.^46^

### Redox Potential Measurements

All redox potential experiments were conducted under anoxic conditions (100% N_2_). Aqueous solutions were purged with N_2_ for at least 2 h before being brought into the glovebox. Open circuit potentials of Fe mineral suspensions were measured in triplicate as a function of both pH and aqueous Fe(II) concentration. Reactors contained 15 mg (1 g L^–1^) of Fe mineral (goethite or magnetite) in 20 mL borosilicate glass vials. To begin the experiment, approximately 15 mL of 50 mM buffer with 25 mM KCl as a background electrolyte was added to the vial along with a small Teflon-coated magnetic stir bar. Solutions were buffered with 2-(*N*-morpholino) ethanesulfonic acid (MES) for pH 5.5 to 6.5, 3-morpholinopropane-1-sulfonic acid (MOPS) for pH 7 to 7.5, and 4-(2-hydroxyethyl)-1-piperazineethanesulfonic acid (HEPES) for pH 8 to 10. Buffers were preadjusted to the desired pH using 1 and 5 M KOH. After buffer addition, aqueous Fe(II) was added from an FeCl_2_ stock and the initial Fe(II) concentration was sampled by filtering the aqueous solution with a 0.22 μm filter. Fe(II) concentrations were measured with the 1,10 phenanthroline method.^47^ Fe mineral suspensions were stirred for 20 min, and a final Fe(II)_aq_ concentration was measured. For experiments where an electron transfer mediator was used, a 10 μM mediator was added to the mineral suspension as the oxidized form of the redox mediator.

To measure the open circuit potential of the solution, we used Pt ring combination redox electrodes (Mettler Toledo, InLab Redox Probe part # 51343200) and a potentiostat for data recording. All measurements were made in reference to the Ag|AgCl electrode (3 M KCl) and then converted to the standard hydrogen electrode (SHE, *E*_H_ +208 mV). The activity for each point was calculated from the ionic strength and experimental conditions using the extended Debye–Hückel equation.^48^ Open-circuit potentials were collected at 2 second intervals for 15 minutes in stirred Fe mineral suspensions after 20 minutes of equilibration. Potentials were measured after 15 minutes of equilibration as we observed limited change (<1 mV change over 1 min) after that time under most experimental conditions. We collected longer term data over 2 weeks and found that there was no substantial difference in the measured potentials after 2 weeks of equilibration between added Fe(II) and magnetite particles (data not shown).

To evaluate whether the buffer was influencing our potential measurements, we measured potentials for buffer concentrations ranging from 0 to 500 mM MOPs in 1 g/L magnetite with ∼1 mM Fe(II) and ∼pH 7.1 (Table S2). Measured potentials among the five MOP concentrations of 0, 1, 5, 50, and 500 mM (run in triplicate) were −237 ± 19 mV (less than 10% error). As expected, however, the pH, Fe(II) sorption, and ionic strength varied and error decreased to ∼5% once differences in ionic strength and pH were accounted for (−237 ± 12 mV). In between measurements, the electrode was rinsed with DI water, dried with a Kimwipe, and resubmerged in storage solution (3 M KCl) until the next sample. Electrodes were periodically polished with alumina, sonicated, and rinsed with DI water before reuse.

To evaluate whether an electron transfer mediator is necessary to measure equilibrium redox potentials, we compared redox potentials of magnetite/Fe(II)_aq_ suspensions with and without several different electron transfer mediators (10 μM). Mediators tested included anthraquinone-2-carboxylic acid, indigo disulfonate, indigo tetrasulfonate, hexaammine ruthenium, anthraquinone-2,6-disulfonate, and anthraquionone-2-sulfonate. Mediators were chosen to span a wide range of redox potentials (from approximately +200 to ∼−400 mV).^32,35,49^ Additionally, we periodically conditioned our electrodes with 1 M HCl for at the least 1 h to remove any impurities at the electrode surface.

Electrodes were validated daily against a purchased redox standard solution (470 ± 10 mV) (Hanna Instruments, HI7022L), and multiple Pt redox electrodes (Mettler Toledo) were used interchangeably. The variation among potentials measured in the same reactors with different electrodes was ≤12 mV. To validate that we could accurately and reproducibly measure redox potentials, we measured redox potentials for goethite equilibrated with aqueous Fe(II) as previous potential measurements have been shown to agree closely with potentials calculated from thermodynamic data.^32,35^ We were able to reproduce the expected *E*_H_ vs pH slope −177 mV for the goethite|Fe(II)_aq_ couple and an *E*_H_^o^ value within the range of expected values (Figure S1 and Table S3).

## Results and Discussion

### Effect of Mediators on Potential Measurements

**Figure 1:**
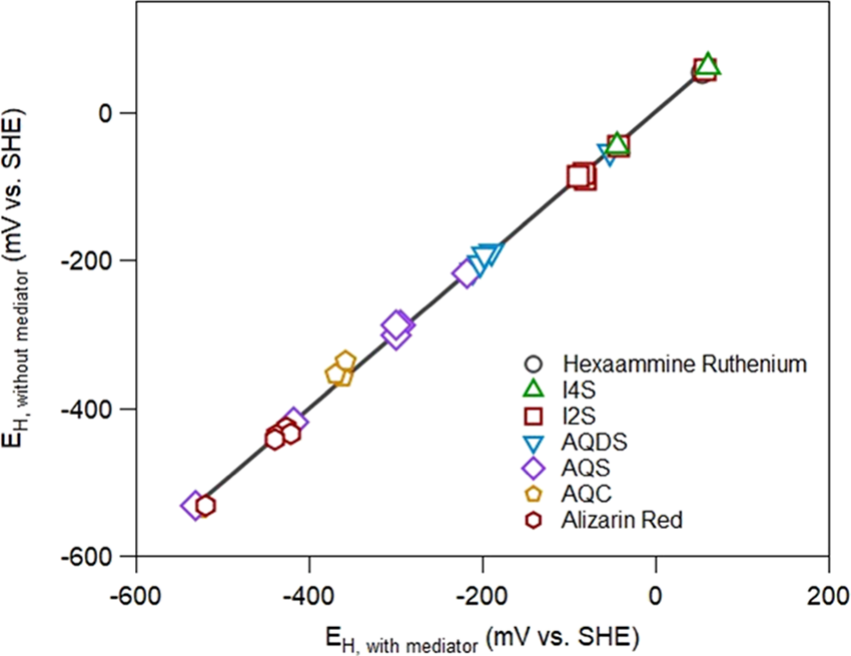
Comparison between redox potential measurements of magnetite|Fe(II)_aq_ suspensions with and without the addition of various mediators. The mediators chosen for our experiment were hexaammine ruthenium, indigo tetrasulfonate, indigo disulfonate, anthraquinone-2,6-disulfonate (AQDS), anthraquinone-2-sulfonate (AQS), anthraquinone-2-carboxylic acid (AQC), and alizarin red. The solid line represents the linear regression *y* = (1.001 ± 0.01) × *x* + (1.86 ± 2.30) for *n* = 30 with *R*^2^ = 0.998. Experimental conditions: 1 g L^–1^ magnetite, 15 mL of 50 mM buffer (MES for pH 5.5–6, MOPS for pH 7 and 7.5, and HEPES for pH 8–9), 25 mM KCl, and 1 mM Fe(II).

To assess whether an electron transfer mediator was necessary to measure an equilibrium potential in magnetite suspensions with added aqueous Fe(II), we measured the potentials of magnetite suspensions over a pH range of 5.5 to 9.0 in the presence and absence of various mediators (Figure 1). We observed near perfect agreement between potentials measured with and without electron mediators (slope of 1.001 ± 0.01, *n* = 30, and *R*^2^ = 0.998), suggesting that mediators were not necessary to reach equilibrium under our experimental conditions, consistent with other’s observation of rapid equilibration of nanomagnetite particles in suspension.^36^ While electron transfer mediators have been previously needed to measure equilibrium potentials for several Fe(III) oxide|Fe(II)_aq_ couples.^35,50−54^ the small particle size (∼11 nm),^34^ solid loading, robust mixing, and/or high conductivity of magnetite^3,29,30^ are likely creating conditions favorable to rapid equilibration (i.e., minimizes suspension effect^55,56^). Additional work will be needed to evaluate whether the rapid equilibration we observed here is more broadly applicable over a range of experimental designs and geochemical conditions.

### Effect of pH and Aqueous Fe(II) on Magnetite Potentials

**Figure 2:**
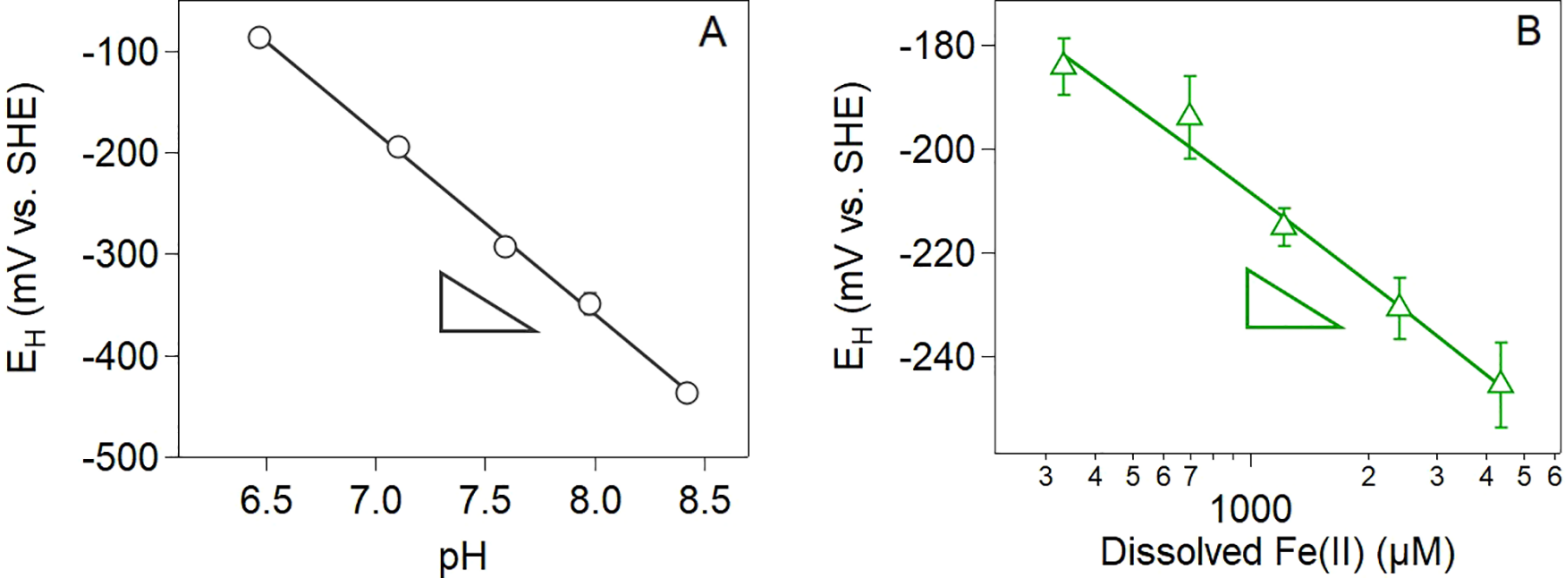
Redox potentials measured for stoichiometric magnetite (*x* = 0.5) suspensions as a function of (A) pH and (B) aqueous Fe(II) concentration. The solid gray line is *E*_H_ = (1075 ± 29) + (−179 ± 4) × pH for *n* = 5 with *R*^2^ = 0.999. The solid green line is *E*_H_ = (−32 ± 13) + (−54 ± 4) × log(Fe(II)) for *n* = 5 with *R*^2^ = 0.984. Experimental conditions: 1 g L^–1^ magnetite (*x* = 0.5), 50 mM buffer (MES for pH 5.5 and 6, MOPS for pH 7, and HEPES for pH 8 and 9) with 25 mM KCl as the background electrolyte. Error bars may be within the size of the marker and represent the standard deviation calculated from triplicate reactors.

To evaluate the effect of pH on magnetite potentials under reducing conditions, we measured potentials for stoichiometric magnetite (*x* = Fe(II)/Fe(III) = 0.5) with 1 mM Fe(II) over a pH range of 6.5 to 8.5 (without electron transfer mediators). We observed Nernstian behavior with an *E*_H_ vs pH slope of −179 ± 4 mV (*n* = 5 and *R*^2^ = 0.999) (Figure 2A). Redox potential measurements on a second batch of synthesized stoichiometric magnetite were found to be reproducible with a near identical slope of −180 ± 6 mV (Figure S2). Surprisingly, the *E*_H_ vs pH slope deviated substantially from the theoretical −236 mV slope for the magnetite|Fe(II)_aq_ redox couple, as well as the −59 mV slope for the maghemite|magnetite and hematite|magnetite redox couples (Table S3). These redox couples have previously been used to interpret magnetite redox potentials measured with Pt redox probes and a modified powder disc electrode.^15,17,32^ Instead, the −179 mV slope measured here closely agrees with the −177 mV slope expected for several Fe(III) oxide|Fe(II)_aq_ redox couples, including Fe(OH)_3(s)_, maghemite, lepidocrocite, hematite, and goethite (Table S3). Interestingly, in the absence of added Fe(II), similar potentials were measured for nanomagnetite below pH of 7.0 and over a range of magnetite stoichiometries.^36^ From data presented in Figure 5 of this work, we estimated an *E*_H_ vs pH slope of −140 ± 13 mV in the absence of Fe(II) compared to an *E*_H_ vs pH slope of −179 ± 4 mV measured here with added Fe(II) (Figure S3). The similar potentials with and without added Fe(II), as discussed in more detail below, are most likely caused by magnetite dissolution and the release of Fe(II) to solution at lower pH values (<7.0) as suggested by Jungcharoen et al. as well.^36^

To evaluate the effect of added aqueous Fe(II) on magnetite potentials, we held the pH constant at 7.1 and measured potentials over a range of Fe(II)_aq_ concentrations from ∼0.2 to 10 mM (Figure 2B). The effect of Fe(II) on *E*_H_ also displayed Nernstian behavior with an *E*_H_ vs Fe(II) slope of −54 ± 4 mV (*n* = 5 and *R*^2^ = 0.984). Our measured *E*_H_ vs Fe(II) slope of −54 mV is within error of the expected slope of −59 mV for several Fe(III) oxide|Fe(II)_aq_ redox couples. The close agreement of both the *E*_H_ vs pH and *E*_H_ vs Fe(II) slopes with Fe(III) oxide|Fe(II)_aq_ redox couples provides compelling evidence that one (or several) of the Fe(III) oxide|Fe(II)_aq_ redox couples (i.e., Fe(OH)_3(s)_, maghemite, lepidocrocite, hematite, or goethite) contributes to poising the potentials in our aqueous Fe(II) and magnetite suspensions (Table S3).

**Figure 3:**
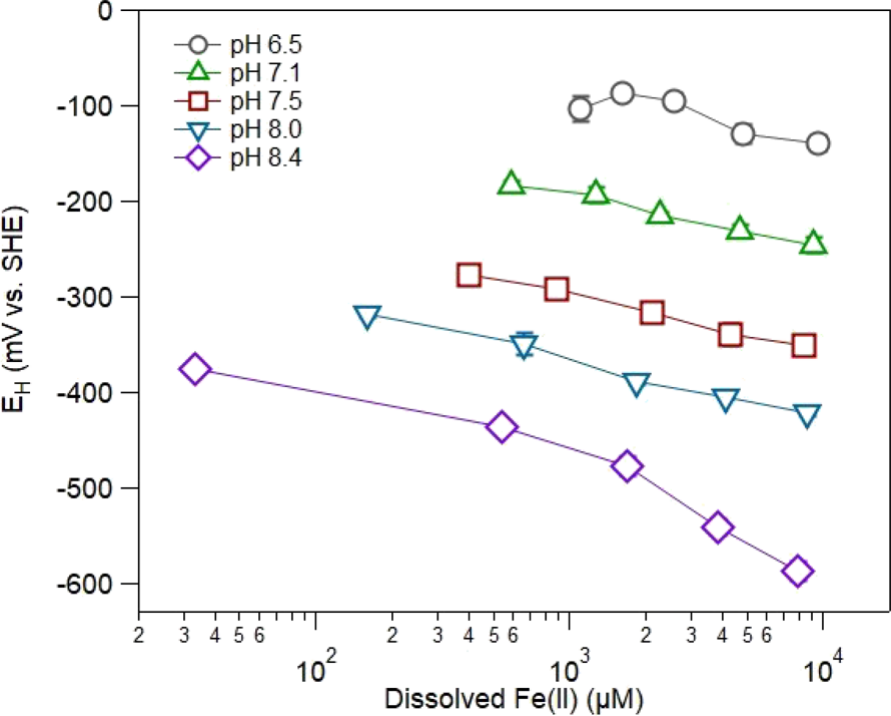
Redox potentials measured for magnetite (*x* = 0.5) suspensions over a range of pH conditions as a function of aqueous Fe(II). Experimental conditions: 1 g L^–1^ magnetite (*x* = 0.5) and 50 mM buffer (MES for pH 6.5, MOPS for pH 7 and 7.5, and HEPES for pH 8 and 8.5) with 25 mM KCl as the background electrolyte. Error bars for measured potentials may be within the marker size and represent the standard deviation calculated from triplicate reactors.

To further narrow down what contributes to poising the potential in the magnetite suspensions, we measured potentials over a wider range of pH values and aqueous Fe(II) concentrations (Figure 3). We observed reasonably linear relationships between measured potentials and aqueous Fe(II) concentrations over a range of pH conditions (6.5 to 8.5) with the expected trend of lower potentials as pH and aqueous Fe(II) increased (*R*^2^ varied from 0.730 to 0.991) (Table S4). The measured potentials, however, begin to deviate from a clear linear trend at the lowest pH of 6.5 and the highest pH of 8.5. Two different processes likely contribute to the deviation from linear behavior at low and high pH values. At the highest pH of 8.5, the saturation index with respect to Fe(OH)_2(s)_ is greater than zero when the initial aqueous Fe(II) concentration is above 1 mM, and we expect FeOH_2(s)_ precipitation to occur (Figure S4). At the lower pH values, the concentration of aqueous Fe(II) increased over time, indicating that net dissolution of Fe(II) from magnetite occurred consistent with previous observations (Figure S5).^16,36,57^

To estimate an *E*_H_^o^ value, we excluded the data points where precipitation was expected (red markers in Figure S4), as well as the pH 6.5 data points where substantial dissolution was measured (Figure S5), and then performed a multivariate regression using eq 7 with both the pH and Fe(II) slopes held constant at the theoretical values of −59 and −177 mV estimated from Figure 2.
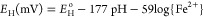
7

This restricted set of measurements (i.e., omitting data where ferrous hydroxide precipitation was expected, and pH 6.5) results in an estimated *E*_H_^o^ of 857 ± 8 mV (*n* = 13, χ^2^ = 2147 reduced χ^2^ = 215) (Figure S6A and Table S5). We assessed how removing these points influences our *E*_H_^o^ estimates by adding each of these datasets back into the regression. When the pH 6.5 data were added back, we estimated an *E*_H_^o^ value of 861 ± 8 mV (*n* = 18, χ^2^ = 4244, reduced χ^2^ = 283) (Figure S6B). When the seven points (where FeOH_2(s)_ precipitation was expected) were included (red markers in Figure S4), we estimate an *E*_H_^o^ value of 850 ± 13 mV (*n* = 25, χ^2^ = 24,239, reduced χ^2^ = 1102) (Figure S6C). The estimated *E*_H_^o^ values vary by only 11 mV among the three datasets, however, as it is clear that dissolution and precipitation occur under the low and high pH conditions we chose an *E*_H_^o^ of 857 ± 8 mV estimated from the fully restricted data (i.e., omitting data where ferrous hydroxide precipitation was expected, and pH 6.5) as our best estimate.

**Figure 4:**
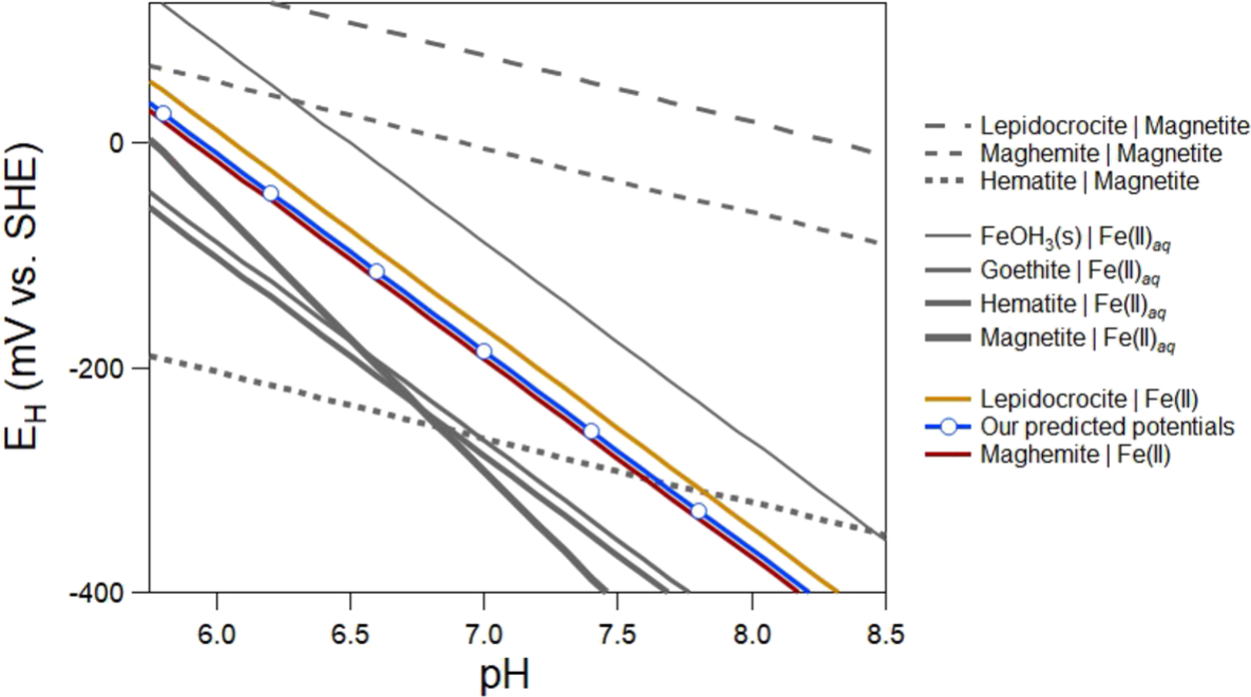
Comparison of theoretical *E*_H_ vs pH lines for various Fe(III)–Fe(II) redox equilibrium couples with the blue predicted potentials based on our estimated *E*^o^ of 855 mV and eq 8.

We compared the theoretical *E*_H_ vs pH lines for the various Fe(III)–Fe(II) equilibria with our predicted potentials using an estimated *E*_H_^o^ of 857 ± 8 mV in an *E*_H_ vs pH diagram (Figure 4). The plot provides a useful illustration of how the various Fe(III)–Fe(II) equilibria compare to the redox potentials we measured for the magnetite–Fe(II)_aq_ suspensions. Specifically, it provides a compelling visual to see how different the *E*_H_ vs pH lines for three solid–solid equilibria (maghemite|magnetite, hematite|magnetite, and lepidocrocite|magnetite) are in both magnitudes of the potentials and the slopes. The diagram also illustrates that four of the Fe(III) oxide|Fe(II)_aq_ redox couples (i.e., Fe(OH)_2_(s), goethite, hematite, and magnetite) are ∼100 mV or more off in magnitude from the measured potentials. The measured potentials agree most closely with the *E*_H_ vs pH lines of maghemite|Fe(II)_aq_ and lepidocrocite|Fe(II)_aq_ redox couples. However, perhaps more compelling is how different the potentials are from several of the other Fe(III)–Fe(II) equilibria often used to interpret redox potentials.

Our estimated *E*_H_^o^ of 857 ± 8 is within error of a theoretical *E*_H_^o^ value of 855 mV for the maghemite|Fe(II)_aq_ couple and is also reasonably close to a theoretical *E*_H_^o^ value of 882 mV expected for the lepidocrocite|Fe(II)_aq_ couple. Our result suggests that maghemite|Fe(II)_aq_ is an important component in poising the potential in our experiments. For stoichiometric magnetite, pH 6.5 to 8.0 with and without added Fe(II)_aq_, our measured potentials are consistent with maghemite|Fe(II)_aq_ equilibria as described by eq 8.
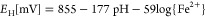
8

The maghemite|Fe(II)_aq_ equilibrium being the best predictor of our observed potentials is consistent with our and others’ spectroscopic work that observed oxidation of Fe(II) in the presence of magnetite to either maghemite or a mixture of low stoichiometry magnetites.^4,23,41^

A third line of evidence supporting maghemite and/or lepidocrocite comes from a rather remarkable LFER developed to model nitroaromatic reduction rates by different Fe minerals reacted with aqueous Fe(II).^32^ During the initial LFER analysis, magnetite was a clear outlier on the linear LFER based on an *E*_H_ estimated from the magnetite|Fe(II)_aq_ couple. When the *E*_H_^o^ for lepidocrocite|Fe(II)_aq_ was used to estimate *E*_H_ instead of magnetite|Fe(II)_aq_, the magnetite rate data fell in line with the observed LFER trend, suggesting that lepidocrocite|Fe(II)_aq_ was a major contributor to poising the potential in those experiments. The maghemite and lepidocrocite *E*_H_^o^ values are, however, close enough (855 and 882 mV) that using the *E*_H_^o^ for maghemite|Fe(II)_aq_ would have achieved a similar effect. It is also, however, possible that lepidocrocite was forming in the presence of the nitroaromatic compound as previous spectroscopic work has identified lepidocrocite forming when nitrobenzene was added to magnetite reacted with Fe(II).^23^ Taken together, our measured potentials combined with previous spectroscopic^4,23,41^ and LFER^32^ results provide three independent lines of evidence that under circum-neutral, reducing conditions where Fe(II)_aq_ is present, magnetite potentials may be reasonably estimated by the maghemite|Fe(II)_aq_ redox couple as given in eq 8.

### Potential Measurements without Added Fe(II)

**Figure 5:**
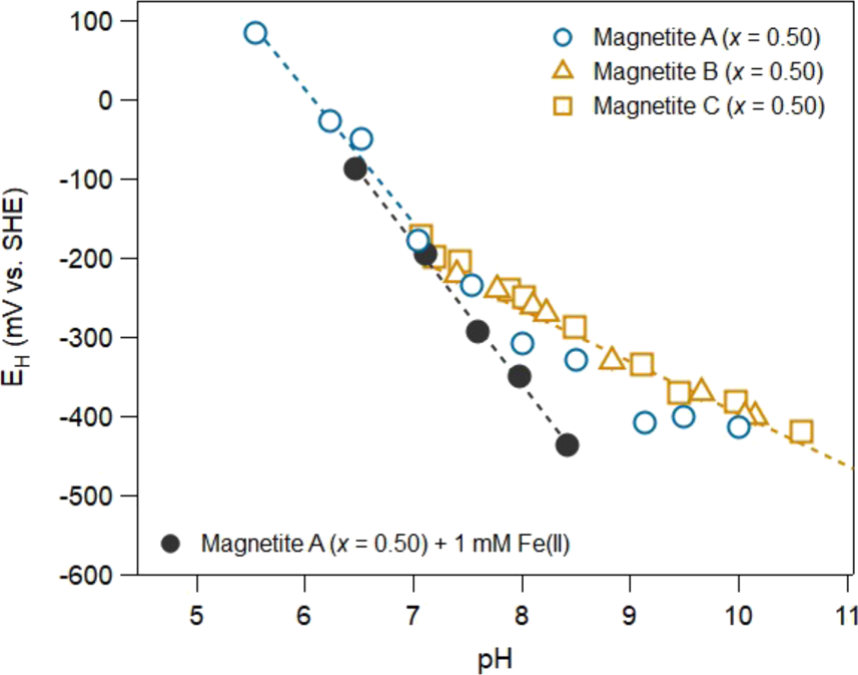
Comparison or redox potentials for magnetite suspensions with and without added aqueous Fe(II) over a range of pH conditions. The dashed lines represent linear fits. Magnetites A, B, and C represent three different batches of stoichiometric magnetite, and each letter dataset was collected by a different person in our lab. Experimental conditions: 1 g L^–1^ magnetite (*x* = 0.5) and 50 mM buffer (MES for pH 6.5, MOPS for pH 7 and 7.5, and HEPES for pH 8 and 8.5) with 25 mM KCl as the background electrolyte. Error bars for measured potentials may be within the marker size and represent the standard deviation calculated from triplicate reactors.

To evaluate how potentials shifted in the magnetite suspensions when no aqueous Fe(II) was added, we measured *E*_H_ as a function of pH for stoichiometric magnetite without added Fe(II) (Figure 5). At pH values below 7.0, the potentials agreed with those measured in the presence of Fe(II) and had an *E*_H_ vs pH slope of −170 ± 19 mV (*n* = 4 and *R*^2^ = 0.976) consistent with the −179 ± 4 mV slope we observed with 1 mM added Fe(II). The similar potentials and effect of pH with and without added Fe(II) below pH 7.0 are consistent with previous work^36^ and suggest that maghemite|Fe(II)_aq_ contributes to poising the potential at low pH regardless of whether Fe(II) is added or not to the magnetite suspensions. Similar potentials at low pH values with and without added Fe(II) are perhaps not all that surprising as below pH 7.0, we and others^36^ measure the release of Fe(II) into the aqueous phase from magnetite dissolution and ultimately the potentials measured are likely being set by the equilibria controlling the ratio of aqueous Fe(II) to Fe(III) in solution (Figure S7).^32,35^

At pH values above 7.0, however, we observed a slope of −66 ± 3 mV (*n* = 26 and *R*^2^ = 0.937) which is markedly different from the −179 ± 4 mV observed with 1 mM added Fe(II) (Figure 5). Note that we included potential measurements made previously by our group for magnetite suspensions in the absence of Fe(II) measured both spectrophotometrically^38^ and electrochemically (magnetite B and magnetite C in Figure 5). An *E*_H_ vs pH slope of −66 ± 3 mV is close to the −59 mV value expected for several solid|solid Fe redox couples, such as maghemite|magnetite, hematite|magnetite, and goethite|magnetite (Table S3). At the higher pH values, a shift from maghemite|Fe(II)_aq_ poising the potential is not surprising given that negligible aqueous Fe(II) was released (Figure S7).

A similar shift in slope at higher pH values (without added Fe(II)) has been previously observed although the potentials were significantly more positive than what we measured here.^36^ A maghemite–magnetite mixing model was used to describe the measured potentials, and the model agreed well with their potentials below pH 7. Above pH 7, however, the solid mixing model does not match their measured potentials over a range of magnetite stoichiometries, nor does it match our stoichiometric magnetite results (Figure S3). It was suggested that the potentials observed at high pH did not match the maghemite–magnetite mixing model because potentials measured at the higher pH values are unreliable as Fe(II) is retained in the solid with little released to solution.^36^ In their work, an electrode with a much smaller contact area was used and may explain the variability observed in their potentials above pH 6.0. Here, we observed consistent potentials and *E*_H_ vs pH slopes among three different datasets collected by three different people using different methods, including two methods with mediators and one without. This level of reproducibility and robustness for our potential measurements above pH 7.0 suggests that it is possible to make reliable *E*_H_ measurements even with low Fe(II) concentrations imposed by the high pH (Figure 5).

### Environmental Implications

Magnetite forms in soils and sediments under reducing conditions, has been shown to reduce several contaminants, and has even been suggested to contribute to abiotic natural attenuation observed in large chlorinated solvent plumes.^3−5,13−25,58,59^ We measured equilibrium potentials for magnetite–aqueous Fe(II) suspensions and found that they are well-described by the maghemite–Fe(II)_aq_ redox couple under circum-neutral, reducing conditions. The measured potentials were highly reproducible, clearly Nernstian, and closely aligned with one Fe(III)_oxide_|Fe(II)_aq_ couple. These results were rather surprising as a wide range of potentials have been reported in the literature with little consensus on how to best measure potentials (i.e., electrode design and configuration), which conceptual model to apply (e.g., solid–solid solution or interfacial electron transfer), and which Fe(III)–Fe(II) equilibria to consider. Our work addresses this significant ambiguity and knowledge gap by providing a compelling case that the potential is being poised by the oxidation of Fe(II) to maghemite consistent with previous Mössbauer spectroscopy results.^23^

The implications of being able to estimate accurate redox potentials of magnetite–Fe(II) suspensions are important for understanding and predicting the role of magnetite in contaminant fate. For example, having a single, simple redox reaction to predict *E*_H_ values in magnetite suspensions provides a promising approach for predicting rates of contaminant reduction by developing LFERs such as the ones previously used to successfully predict rates of nitroaromatic reduction in Fe mineral suspensions and carbon tetrachloride by plume sediments.^9,32^ Our work also raises the interesting question of how magnetite redox potentials will vary beyond the circum-neutral, reducing conditions we studied here and how the variable stoichiometry of magnetite will influence potentials. Magnetite stoichiometry has been shown to dramatically influence rates and/or extent of nitroaromatic, U, and Hg reduction,^4,5,24^ and while there is some evidence that stoichiometry affects measured redox potentials,^34,36,41^ there is significant discrepancy in the measured potentials which our^,^ experimental approach may be able to resolve. Understanding how potentials vary with magnetite stoichiometry, as well as under more complex conditions (such as when Fe(OH)_2_(s) precipitates),^60^ may allow us to quantitatively predict the effect of magnetite stoichiometry and secondary mineral precipitation on contaminant reduction by magnetite. Finally, an improved understanding of other Fe(II)–Fe(III) oxide redox reactions has led to a deeper understanding of the mechanism controlling bacterial Fe reduction using electron transfer mediators.^61^ Our results may provide improved information behind the biological mechanisms of iron reduction and oxidation, similar to those that have been observed with reversible biological magnetite redox cycling in “biogeobatteries”.^62^

## Supporting Information Available

Thermodynamic calculations, model fits, literature estimations, and tabulated experimental and literature data (PDF)
