# Supplementary material for: Redox Potentials of Magnetite Suspensions under Reducing Conditions
Source: Environ Sci Technol. 2022 Nov 17;56(23):17454–61. doi: 10.1021/acs.est.2c05196 (PMC9730839; doi:10.1021/acs.est.2c05196)
Supplement: Supplementary file 1 — es2c05196_si_001.pdf [file es2c05196_si_001.pdf]

Supporting Information

Redox potentials of magnetite suspensions under reducing conditions

Thomas C. Robinson,[†] Drew E. Latta,[†] Johna Leddy[‡], Michelle M. Scherer[†]
†Department of Civil and Environmental Engineering, University of Iowa, Iowa City, Iowa 52242, United States
‡Department of Chemistry, University of Iowa, Iowa City, Iowa 52242, United States

The supporting information contains:

Figures: 8

Tables: 7

Pages: 23

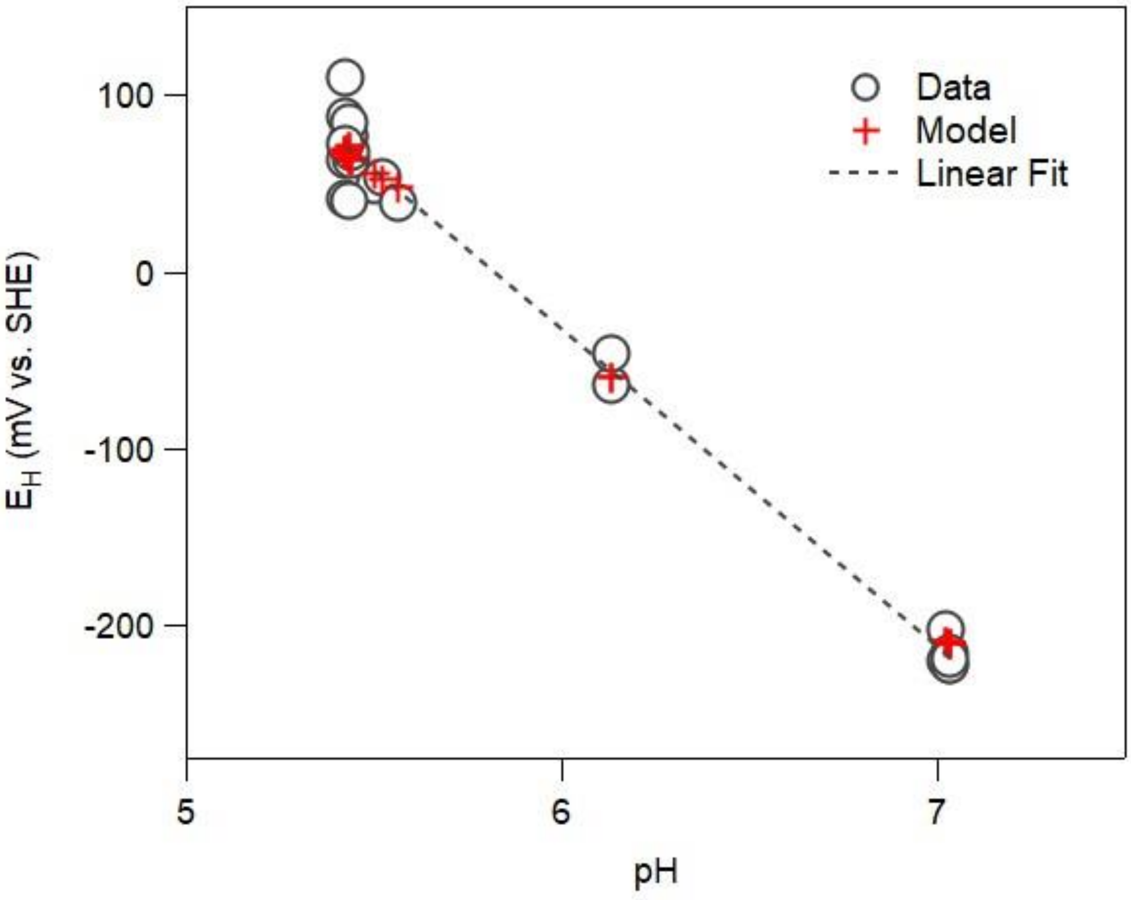

Figure S1. Open circuit potentials for goethite suspensions with added aqueous Fe(II) as a function of pH. The dashed grey line represents a linear regression with a slope of 179 ± 5 (n = 22 and R^2 = 0.987) which closely matches the -177 mV slope expected for the goethite | Fe(II)$_{aq}$ couple. Holding the Fe(II) and pH slopes to Goethite | Fe(II) theoretical values of -59 and -177 gives an estimated E° of 837 ± 7 [E_H = (837 ± 7) – 59 * log (aFe2) – 177 * pH for n = 22 and χ^2 = 5233, reduced χ^2 = 275] which is similar, but slightly higher than the calculated theoretical value of 782 mV for the goethite | Fe(II) redox couple assuming 1 mM aqueous Fe(II) and an activity of 0.553. **Experimental conditions:** 1 g L^{-1} goethite, 1 mM Fe(II), 50 mM Buffer (MES for pH 5.5 and 6, MOPS for pH 7) with 25 mM KCl as a background electrolyte, 10 µM mediator.

Table S1. E_H^o value calculated for selected Fe redox couples from several thermodynamic data sources.

Redox Couple[a]	**NEA[1b] (V)**	Navrotsky et al. 2008[2] (V)	Rickard and Luther 2007[3] (V)	Langmuir 1997[4] (V)	Stumm and Morgan 1996[5] (V)	Robie and Hemingway 1995[6] (V)	E_H^o Average (V)
Maghemite | Magnetite	**0.410**	-[c]	0.438	0.796	-	-	0.548 ± 0.215
Hematite | Magnetite	**0.151**	-	0.008	0.177	0.178	0.152	0.133 ± 0.071
Goethite | Fe(II)	**0.782**	0.771	0.790	0.644	0.670	0.750	0.734 ± 0.062
Magnetite | Fe(II)	**1.078**	-	1.060	0.894	0.895	1.066	0.999 ± 0.095
Lepidocrocite | Fe(II)	**0.882**	0.853	0.985	0.849	-	-	0.892 ± 0.064

a. When not reported the $Fe(II)_{aq}$ and H_2O ΔG values used were the values reported in the NEA database.
b. Bold entries represent the calculated E_H^o values used for this study.
c. Dashes represent E_H^o values that could not be calculated from the information provided in the chosen reference.

Table S2. Effect of MOPS and KCl concentration on measured E_H of 1 g/L magnetite and 0.9 mM Fe(II).

[MOPS], mM	[KCl], mM	pH	$[Fe(II)]_f$, mM†	E_H measured (mV)	E_H from Eq. 7‡ (mV)
**Vary MOPS**					
0	25	7.26 ± 0.03[a]	0.781 ± 0.106[a]	-254 ± 12.8[a]	-232 ± 5.4[b]
1	25	7.19 ± 0.04	0.863 ± 0.023	-255 ± 7.1	-221 ± 7.4
5	25	7.13 ± 0.01	1.02 ± 0.017	-239 ± 2.3	-214 ± 2.4
50[c]	25	7.12 ± 0.01	1.15 ± 0.012	-227 ± 7.8	-209 ± 1.2
500	25	7.14 ± 0.01	1.20 ± 0.012	-210 ± 3.2	-201 ± 2.4
**Vary KCl**					
50	2.5	7.08 ± 0.00	1.09 ± 0.040	-224 ± 3.1	-204 ± 0.9
50[c]	25	7.12 ± 0.01	1.15 ± 0.012	-227 ± 7.8	-209 ± 1.2
50	250	7.05 ± 0.00	1.13 ± 0.043	-208 ± 2.1	-187 ± 1.0

[a] Averages and standard deviations (1σ) for measured values in triplicate reactors

[b] Averages and standard deviation (1σ) for calculated E_H using individual measured Fe(II) and pH values inputted into Eq. 8 in the main text.

[c] These data are the same and are repeated in the Vary [MOPS] and Vary [KCl] to aid the reader.

† Initial [Fe(II)] = 0.907 ± 0.001 mM

‡ Activity coefficients for $Fe^{2+}(aq)$ were computed using the amount of ionized buffer and K^+ added as KOH to adjust the pH, the amount of KCl present, and $FeCl_2$ added in Geochemists Workbench SpecE8 routine. An activity correction was applied by taking the ratio of the condition corrected to the activity coefficient for 50 mM MOPS/25 mM KCl base condition ($\gamma_{Fe(II)}$=0.4284).

Redox potential measurements of goethite suspensions equilibrated with Fe(II). To confirm that we could accurately and reproducibly measure redox potentials, we measured redox potentials for goethite in solution equilibrated with aqueous Fe(II) as previous potential measurements have been shown to agree closely with potentials calculated from thermodynamic data.[7, 8] We observed Nernstian behavior for potentials measured as a function of pH with an E_H vs. pH slope of -179 mV (n = 22 and R^2 = 0.987) which agrees closely with the theoretical slope of -177 mV for the goethite | $Fe(II)_{aq}$ couple (**Table S3**). Since our estimated values for the pH and Fe(II) slope were close to the theoretical value, we assumed the error was experimental and held both the pH and Fe(II) slopes to their theoretical values of -59 and -177 mV, respectively, to estimate an E_H^o value of 837 ± 7 mV (n = 22, χ^2 = 5,233, and reduced χ^2 = 275) (**Figure S1**). Our estimated E_H^o value of 837 ± 7 mV falls within the range of previously published values calculated from thermodynamics which range from 764 to 861 mV[9-11] (**Table S3).**

Table S3. Calculated thermodynamic values for relevant Fe redox couples.

Redox Couple	Half Reaction	NEA - ΔG_{rxn}[a] (kJ mol^{-1})	NEA E_H^o (V)	Fe(II) slope (V)	pH slope (V)
	Solid | Solid				
Goethite | Magnetite<br>α -FeOOH | Fe_3O_4	$3\alpha - \mathrm{FeOOH} + H^+ + e^- \rightarrow Fe_3O_4 + 2H_2O$	-18.38	0.190	-	-0.059
Hematite | Magnetite<br>α - Fe_2O_3 | Fe_3O_4	$3\alpha - \mathrm{Fe_2O_3} + 2\mathrm{H}^+ + 2\mathrm{e}^- \rightarrow 2\mathrm{Fe_3O_4} + \mathrm{H_2O}$	-29.23	0.152	-	-0.059
Maghemite | Magnetite<br>γ - Fe_2O_3 | Fe_3O_4	$3\gamma - Fe_2O_3 + 2H^+ + 2e^- \rightarrow 2Fe_3O_4 + H_2O$	-79.09	0.410	-	-0.059
Lepidocrocite | Magnetite<br>γ - FeOOH | Fe_3O_4	$\boldsymbol{3\gamma FeOOH + H^+ + e^- \rightarrow Fe_3O_4 + 2H_2O}$	-47.36	0.491	-	-0.059
	Solid | Aq. Fe(II)				
Magnetite | $Fe(II)_{aq}$<br>Fe_3O_4 | Fe(II)	$Fe_3O_4 + 8H^+ + 2e^- \rightarrow 3Fe^{2+} + 4H_2O$	-208.00	1.078	-0.089	-0.236
Goethite | $Fe(II)_{aq}$<br>α -FeOOH | Fe(II)	$\alpha - \mathrm{FeOOH} + 3H^+ + e^- \rightarrow Fe^{2+} + 2H_2O$	-75.46	0.782	-0.059	-0.177
Lepidocrocite | $Fe(II)_{aq}$<br>γ - FeOOH | Fe(II)	$\gamma - FeOOH + 3H^+ + e^- \rightarrow Fe^{2+} + 2H_2O$	-85.12	0.882	-0.059	-0.177
Hematite | $Fe(II)_{aq}$<br>α - Fe_2O_3 | Fe(II)	$\alpha - Fe_2O_3 + 6H^+ + 2e^- \rightarrow 2Fe^{2+} + 3H_2O$	-148.41	0.769	-0.059	-0.177
Maghemite | $Fe(II)_{aq}$<br>γ - Fe_2O_3 | Fe(II)	$\gamma - Fe_2O_3 + 6H^+ + 2e^- \rightarrow 2Fe^{2+} + 3H_2O$	-165.03	0.855	-0.059	-0.177
Ferrihydrite | $Fe(II)_{aq}$[b]<br>$Fe(OH)_{3(s)}$ | Fe(II)	$Fe(OH)_{3(s)} + 3H^+ + e^- \rightarrow Fe^{2+} + 3H_2O$	-92.53	0.959	-0.059	-0.177

a. ΔG_{rxn} calculated with thermodynamic values reported in the Nuclear Energy Agency, Chemical Thermodynamics of Iron Volume 1.[1]

b. Values calculated from Water Chemistry Table 11.4[12]

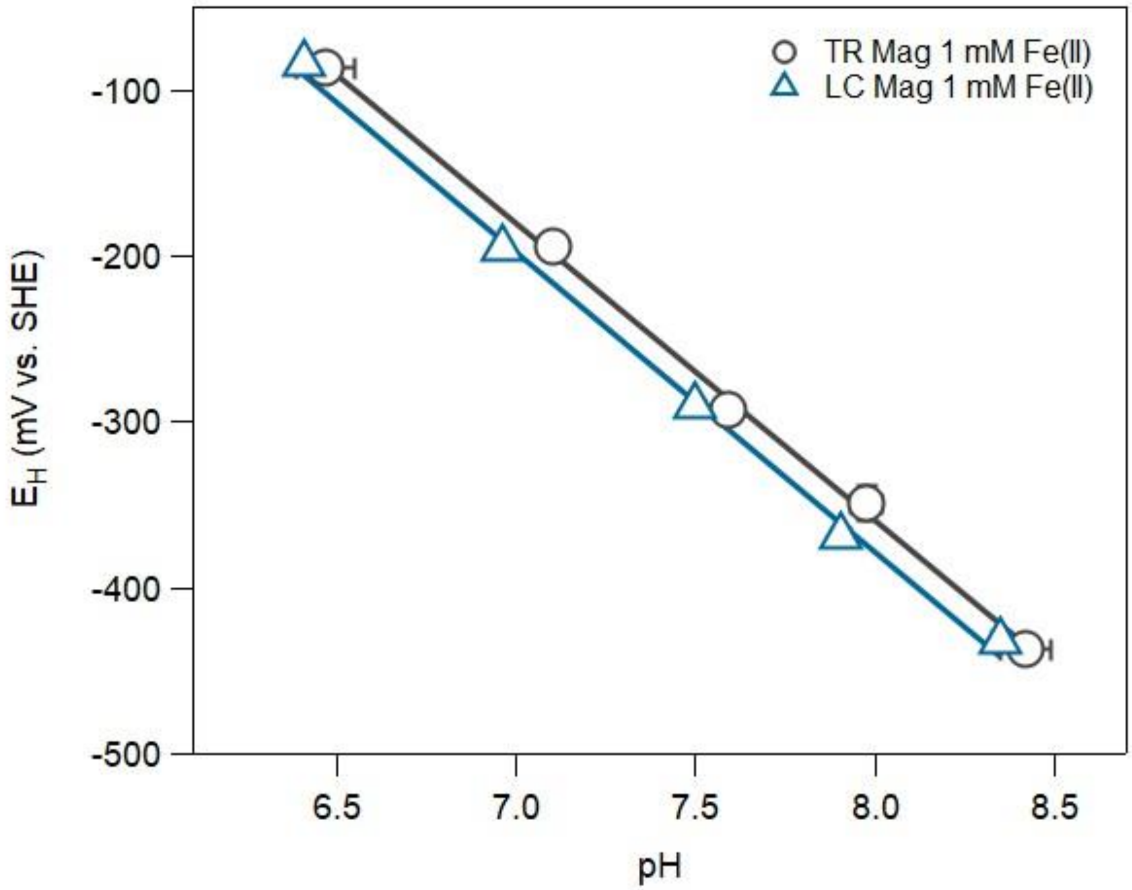

Figure S2. Comparison between the measured redox potentials for two different batches of stoichiometric magnetite synthesized with the same method. **Experimental conditions:** 1 g L^{-1} magnetite, 50 mM buffer (MES for pH 6.5, MOPS for pH 7 and 7.5, and HEPES for pH 8 and 8.5) with 25 mM KCl as a background electrolyte, 1 mM Fe(II). Error bars may be within the size of the marker and represent the standard deviation calculated from triplicate reactors.

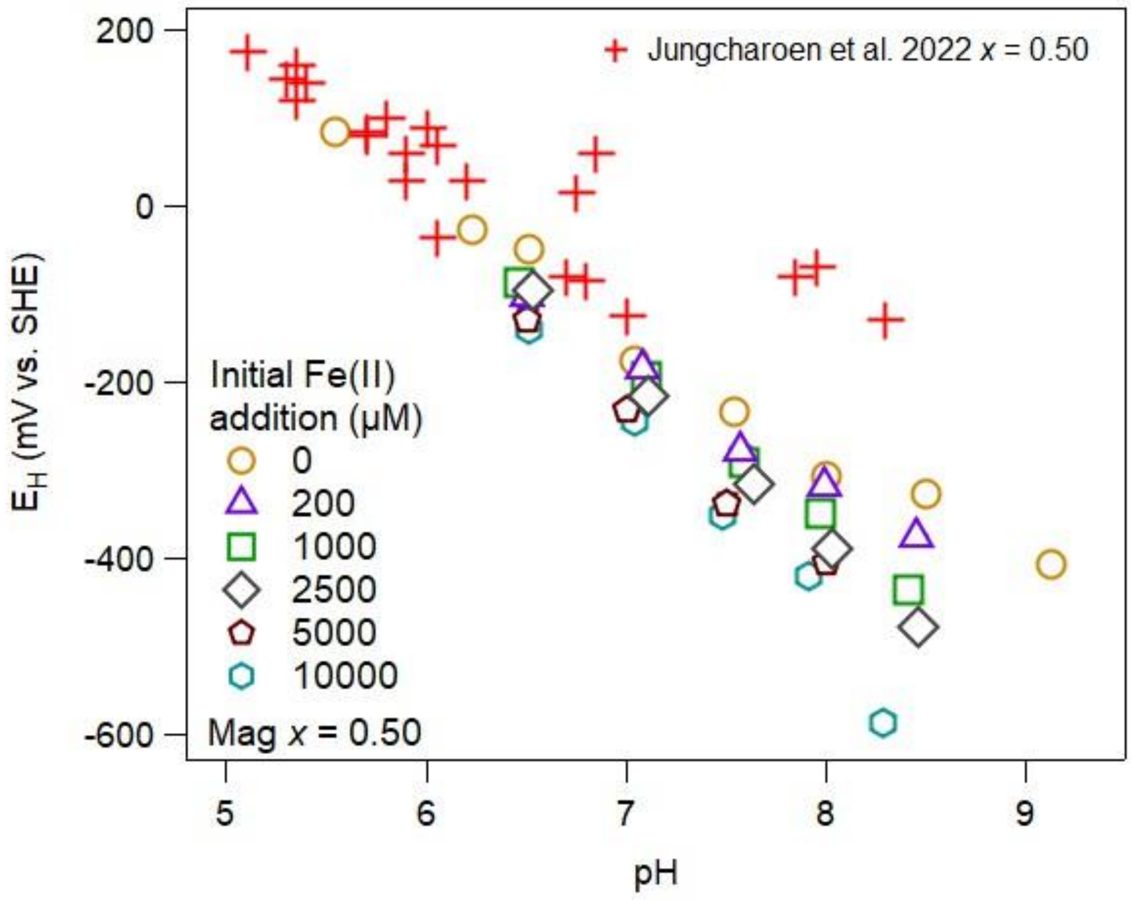

Figure S3. Comparison between estimated potential measurements from Jungcharoen et al. 2022[13] and our results using the data presented in **Figure 3** as a function of pH.

Table S4. Linear regressions for ORP vs. Log(Fe(II)) relationships in **Figure 3**

pH	Intercept	Slope	R^2
6.5	67 ± 63	-51 ± 18	0.730
7	-32 ± 13	-54 ± 4	0.984
7.5	-122 ± 11	-59 ± 3	0.991
8	-180 ± 13	-62 ± 4	0.988
8.5	-225 ± 47	-86 ± 15	0.917

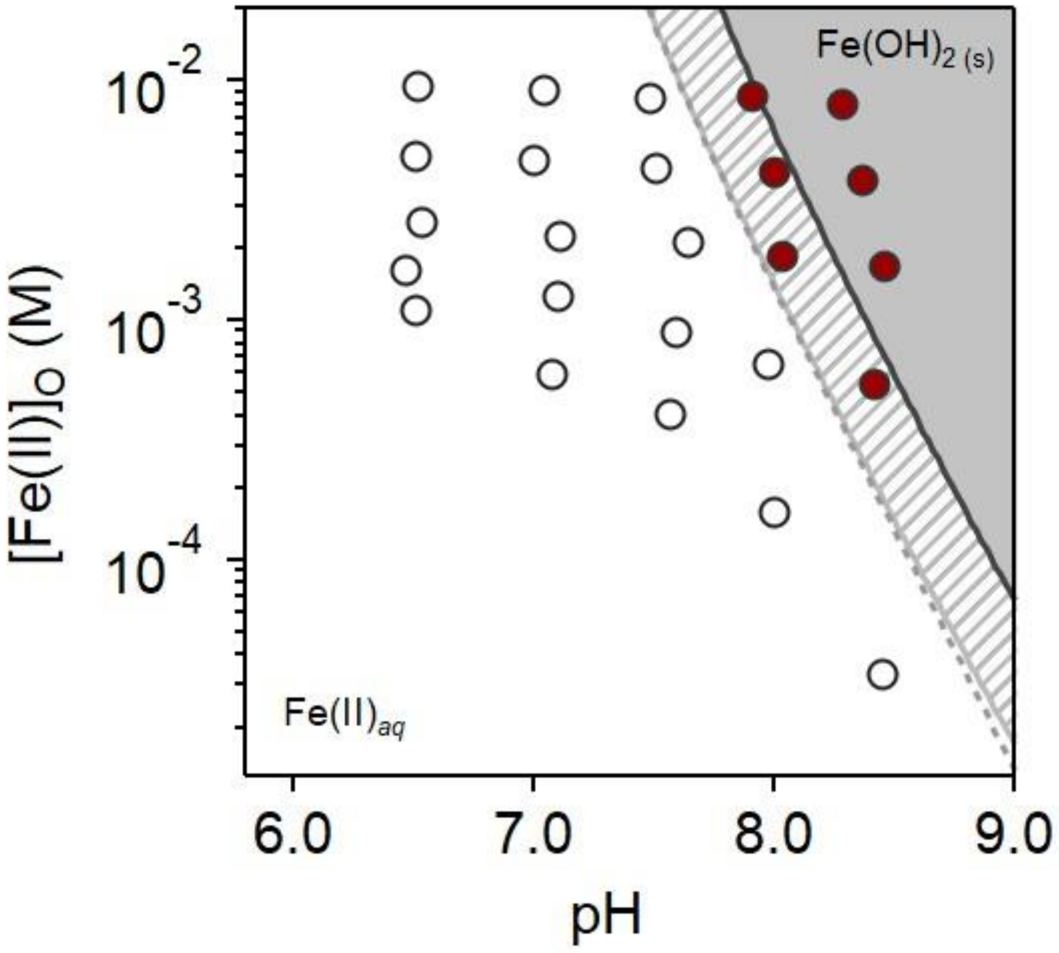

Figure S4. $Fe(OH)_{2(S)}$ solubility diagram with the experimental conditions for our magnetite suspensions overlaid on top. Red markers represent conditions where SI > 0 and $Fe(OH)_2(s)$ is expected to precipitate. The K_{sp} for $Fe(OH)_{2(S)}$ was assumed to range between $10^{-14.51}$ and $10^{-15.11}$ representing more amorphous and more crystalline $Fe(OH)_2(s)$ respectively.[14] SI was calculated from the ion activity product (IAP) of $[Fe^{2+}][OH^-]_2$ and a K_{sp} of -15.11 for crystalline $Fe(OH)_{2(s)}$.[14]

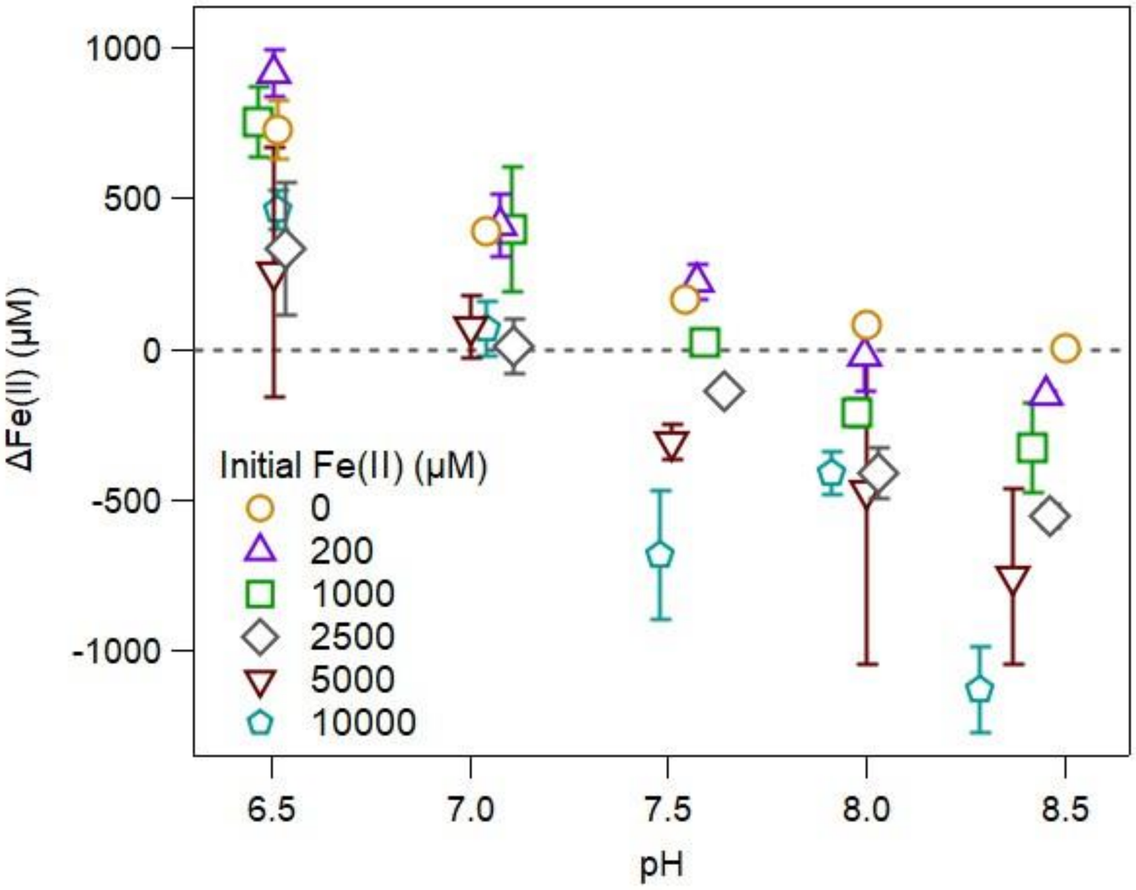

Figure S5. Measured $\Delta[Fe(II)_{aq}]$ in aqueous Fe(II) magnetite suspensions as a function of pH and initial aqueous Fe(II) concentration. $\Delta[Fe(II)_{aq}]$ is defined as the difference between $[Fe(II)_{aq.}]_{final}$ and $[Fe(II)_{aq,}]_{initial}$ measured after 20 minutes of equilibration. The grey dashed line is included to guide the eye and separate the top region where dissolution occurred, and the bottom region where $Fe(OH)_{2(s)}$ precipitation may have occurred. Error bars may be within the size of the marker and represent the standard deviation calculated from triplicate reactors.

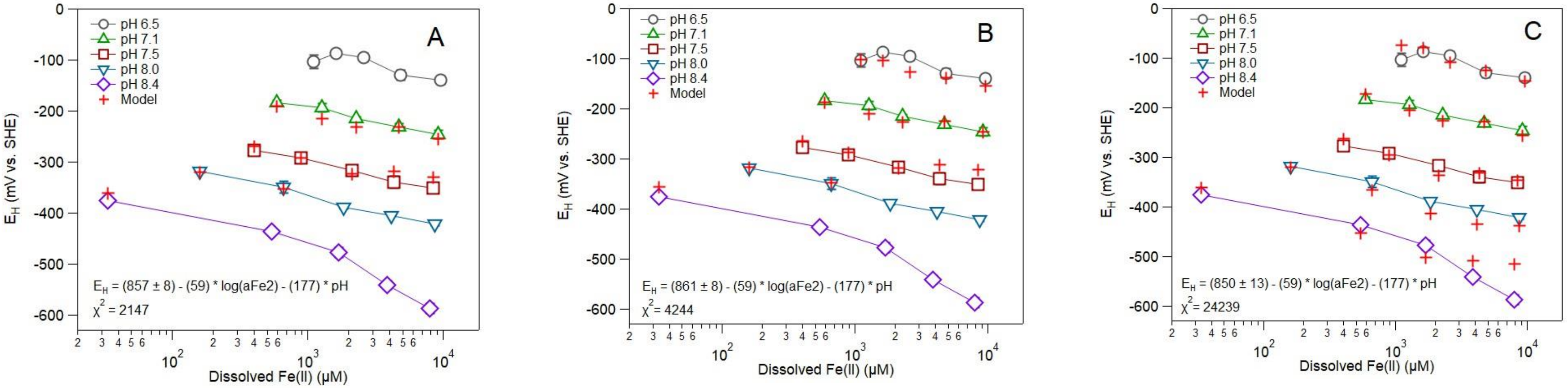

Figure S6. Multivariate regression results superimposed on the data presented in Figure 3. The regression was developed from the Nernst equation where three variable, ORP, pH, and Fe(II) concentration were estimated with three parameters, the Fe(II) slope, the pH slope, and E_H^o.

Table S5. Fitting parameters for multivariate regressions included in **Figure S6**.

Restriction	*n*	E_H^o (mV vs. SHE)	Fe slope	pH slope	χ^2
SI > 0 and pH 6.5 (A)	13	855 ± 8	-59	-177	2275
SI > 0 (B)	18	861 ± 8	-59	-177	4244
All data (C)	25	850 ± 13	-59	-177	24239

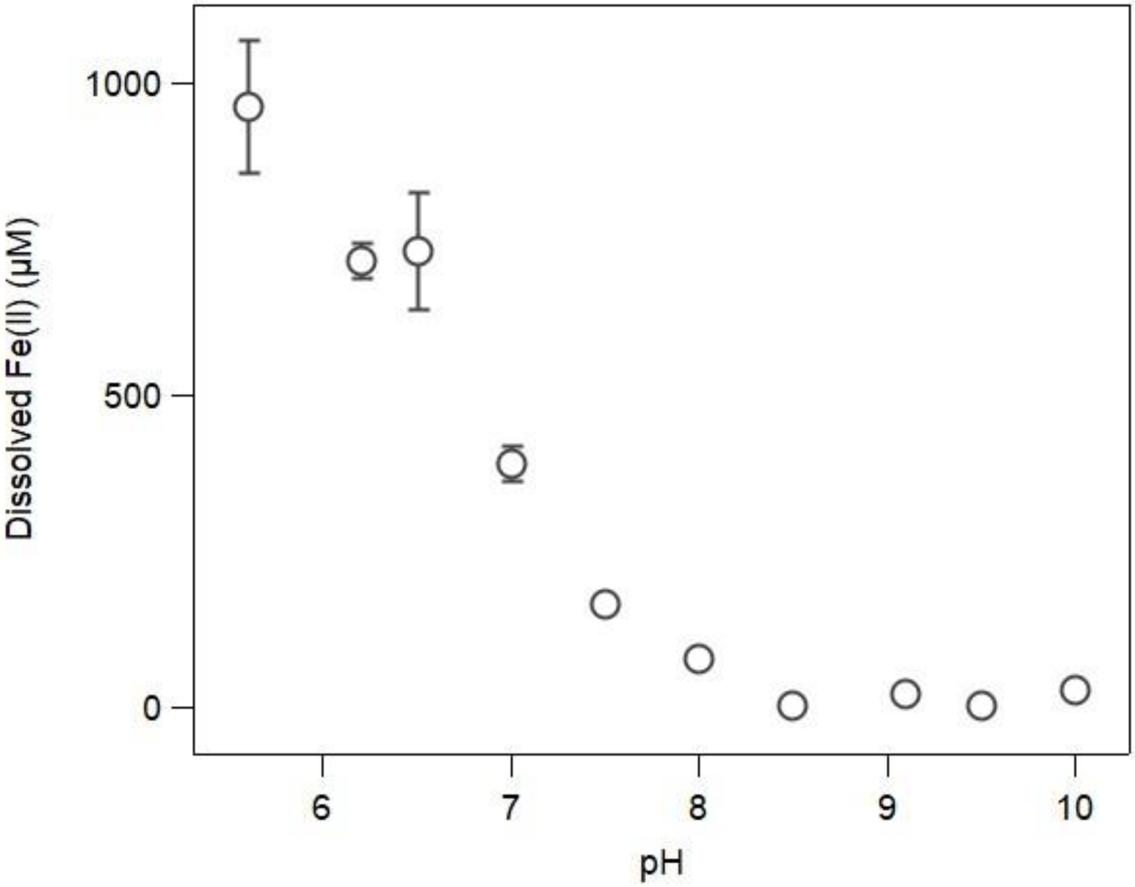

Figure S7. Aqueous Fe(II) measurements made for stoichiometric magnetite suspended with no added Fe(II) as a function of pH. Mineral dissolution is observed for the lower pH conditions (pH 5.5 to 7.5) and is not observed for the higher pH conditions (pH 8.0 to 10.0). Error bars may be inside the size of the marker and represent the standard deviation calculated from triplicate reactors.

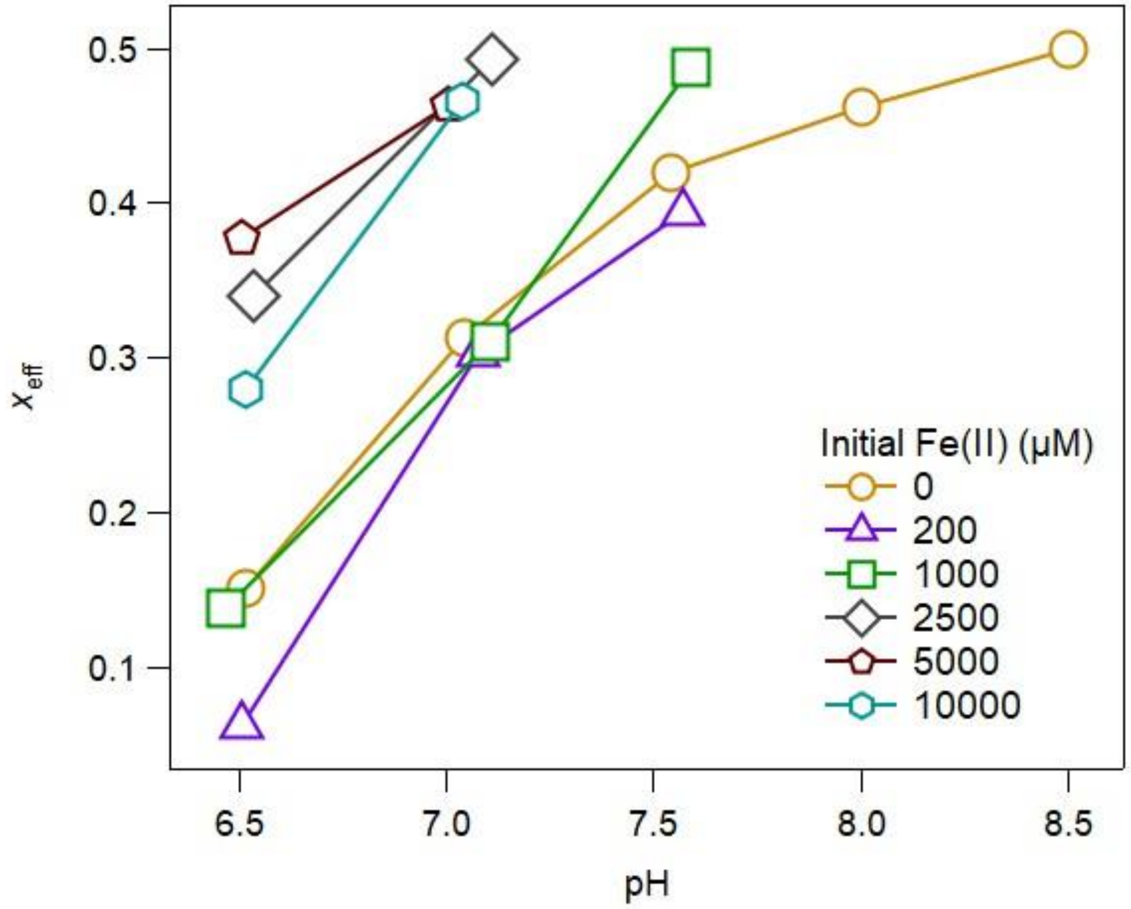

Figure S8. Calculated magnetite stoichiometry as a function of pH and initial Fe(II) concentration. All changes in stoichiometry are caused by mineral dissolution and are estimates based on the difference between aqueous Fe(II) measurements before and after 20 minutes of equilibration. Data points where $Fe(II)_{aq}$ decreased were omitted.

Thermodynamic calculations

Table S6: Gibbs free energies of formation (ΔG_f^o, kJ mol^{-1}) assuming 25°C and 1 atm of pressure.

Term	ΔG_f^o (kJ/mol)
H_2O	-237.14
Fe^{2+}	-90.72
H^+	0
e^-	0
α - FeOOH	-489.54
γ - FeOOH	-479.88
α - Fe_2O_3	-744.45
γ - Fe_2O_3	-727.83
Fe_3O_4	-1012.72

Here, we used a single source of thermodynamic data (i.e., Chemical Thermodynamics of Iron Volume 1[1]) (**Table S6**) to calculate E_H^o values to avoid any inconsistencies.

Solid to Solid

Redox equation for goethite and magnetite

$$\boldsymbol{3\alpha - \mathrm{FeOOH} + H^+ + e^- = Fe_3O_4 + 2H_2O}$$

$$\Delta \mathrm{G^o}_{\mathrm{rxn}} = \Delta G^o{}_{Mag} + 2\Delta G^o{}_{H_2O} - 3\Delta G^o{}_{Gt} - \Delta G^o{}_{H^+}$$

$$\Delta \mathrm{G^o}_{\mathrm{rxn}} = (-1012.72) + 2 * (-237.14) - 3 * (-489.54) - (0)$$

$$\Delta \mathrm{G^o}_{\mathrm{rxn}} = -18.38 \frac{kJ}{mol}$$

$$\Delta \mathrm{G^o}_{\mathrm{rxn}} = -nF * E_H^o$$

$$-18.38 = -(1)(96.485) * E_H^o$$

$$E_H^o = 0.190 \frac{V}{mol}$$

Redox equation for maghemite and magnetite

$$\boldsymbol{3\gamma Fe_2O_3 + 2H^+ + 2e^- = 2Fe_3O_4 + H_2O}$$

$$\Delta \mathrm{G^o}_{\mathrm{rxn}} = 2\Delta G^o{}_{Mag} + \Delta G^o{}_{H_2O} - 3\Delta G^o{}_{Magh} - 2\Delta G^o{}_{H^+}$$

$$\Delta \mathrm{G^o}_{\mathrm{rxn}} = 2 * (-1012.72) + (-237.14) - 3 * (-727.83) - 2 * (0)$$

$$\Delta \mathrm{G^o}_{\mathrm{rxn}} = -79.09 \frac{kJ}{mol}$$

$$\Delta \mathrm{G^o}_{\mathrm{rxn}} = -nF * E_H^o$$

$$-79.09 = -(2)(96.485) * E_H^o$$

$$E_H^o = 0.410 \frac{V}{mol}$$

Redox equation for hematite and magnetite

$$3\alpha Fe_2O_3 + 2H^+ + 2e^- = 2Fe_3O_4 + H_2O$$

$$\Delta G^o{}_{rxn} = 2\Delta G^o{}_{Mag} + \Delta G^o{}_{H_2O} - 3\Delta G^o{}_{Hem} - 2\Delta G^o{}_{H^+}$$

$$\Delta G^o{}_{rxn} = 2*(-1012.72) + (-237.14) - 3*(-744.45) - 2*(0)$$

$$\Delta G^o{}_{rxn} = -29.23\ \frac{kJ}{mol}$$

$$\Delta G^o{}_{rxn} = -nF * E_H^o$$

$$-29.23 = -(2)(96.485) * E_H^o$$

$$E_H^o = 0.1515\frac{V}{mol}$$

Redox equation for lepidocrocite and magnetite

$$3\gamma FeOOH + H^+ + e^- = Fe_3O_4 + 2H_2O$$

$$\Delta G^o{}_{rxn} = \Delta G^o{}_{Mag} + 2\Delta G^o{}_{H_2O} - 3\Delta G^o{}_{Lepid} - \Delta G^o{}_{H^+}$$

$$\Delta G^o{}_{rxn} = (-1012.72) + 2*(-237.14) - 3*(-479.88) - (0)$$

$$\Delta G^o{}_{rxn} = -47.36\frac{kJ}{mol}$$

$$\Delta G^o{}_{rxn} = -nF * E_H^o$$

$$-47.36 = -(1)(96.485) * E_H^o$$

$$E_H^o = 0.491\frac{V}{mol}$$

Solid to Aqueous Fe(II)

Redox equation for Lepidocrocite and Fe(II)

$$\gamma - \mathbf{FeOOH} + \boldsymbol{3H^{+}} + \boldsymbol{e^{-}} = \boldsymbol{Fe^{2+}} + \boldsymbol{2H_2O}$$

$$\Delta \mathrm{G^o}_{\mathrm{rxn}} = \Delta G^o{}_{\boldsymbol{Fe}^{2+}} + 2\Delta G^o{}_{H_2O} - \Delta G^o{}_{Lepidocrocite} - 2\Delta G^o{}_{H^+}$$

$$\Delta \mathrm{G^o}_{\mathrm{rxn}} = (-90.72) + 2 * (-237.14) - (-479.88) - 3 * (0)$$

$$\Delta \mathrm{G^o}_{\mathrm{rxn}} = -85.12 \frac{kJ}{mol}$$

$$\Delta \mathrm{G^o}_{\mathrm{rxn}} = -nF * E_H^o$$

$$-85.12 = -(1)(96.485) * E_H^o$$

$$E_H^o = 0.882 \frac{V}{mol}$$

Redox equation for Goethite and Fe(II)

$$\boldsymbol{\alpha} - \mathbf{FeOOH} + \boldsymbol{3H^{+}} + \boldsymbol{e^{-}} = \boldsymbol{Fe^{2+}} + \boldsymbol{2H_2O}$$

$$\Delta \mathrm{G^o}_{\mathrm{rxn}} = \Delta G^o{}_{\boldsymbol{Fe}^{2+}} + 2\Delta G^o{}_{H_2O} - \Delta G^o{}_{Goethite} - 2\Delta G^o{}_{H^+}$$

$$\Delta \mathrm{G^o}_{\mathrm{rxn}} = (-90.72) + 2 * (-237.14) - (-489.537) - 3 * (0)$$

$$\Delta \mathrm{G^o}_{\mathrm{rxn}} = -75.463 \frac{kJ}{mol}$$

$$\Delta \mathrm{G^o}_{\mathrm{rxn}} = -nF * E_H^o$$

$$-75.463 = -(1)(96.485) * E_H^o$$

$$E_H^o = 0.7821 \frac{V}{mol}$$

Redox equation for hematite and Fe(II)

$$\boldsymbol{\alpha Fe_2O_3 + 6H^+ + 2e^- = 2Fe^{2+} + 3H_2O}$$

$$\Delta G^o{}_{rxn} = 2\Delta G^o{}_{Fe^{2+}} + 3\Delta G^o{}_{H_2O} - 3\Delta G^o{}_{Hem} - 6\Delta G^o{}_{H^+}$$

$$\Delta G^o{}_{rxn} = 2*(-90.72) + 3*(-237.14) - (-744.45) - 2*(0)$$

$$\Delta G^o{}_{rxn} = -148.41\ \frac{kJ}{mol}$$

$$\Delta G^o{}_{rxn} = -nF * E_H^o$$

$$-148.41 = -(2)(96.485) * E_H^o$$

$$E_H^o = 0.769\frac{V}{mol}$$

Redox equation for maghemite and Fe(II)

$$\boldsymbol{\gamma Fe_2O_3 + 6H^+ + 2e^- = 2Fe^{2+} + 3H_2O}$$

$$\Delta G^o{}_{rxn} = 2\Delta G^o{}_{Fe^{2+}} + 3\Delta G^o{}_{H_2O} - \Delta G^o{}_{Magh} - 6\Delta G^o{}_{H^+}$$

$$\Delta G^o{}_{rxn} = 2*(-90.72) + 3*(-237.14) - (-727.83) - 6*(0)$$

$$\Delta G^o{}_{rxn} = -165.03\frac{kJ}{mol}$$

$$\Delta G^o{}_{rxn} = -nF * E_H^o$$

$$-165.03 = -(2)(96.485) * E_H^o$$

$$E_H^o = 0.855\frac{V}{mol}$$

Redox equation for aqueous Fe(II) and magnetite

$$\boldsymbol{Fe_3O_4 + 8H^+ + 2e^- = 3Fe^{2+} + 4H_2O}$$

$$\Delta G^{o}_{rxn} = 3\Delta G^{o}{}_{Fe^{2+}} + 4\Delta G^{o}{}_{H_2O} - \Delta G^{o}{}_{Mag} - 8\Delta G^{o}{}_{H^+}$$

$$\Delta G^{o}_{rxn} = 3 * (-90.72) + 4 * (-237.14) - (-1012.72) - 8 * (0)$$

$$\Delta G^{o}_{rxn} = -208 \frac{kJ}{mol}$$

$$\Delta G^{o}_{rxn} = -nF * E_H^o$$

$$-208 = -(2)(96.485) * E_H^o$$

$$E_H^o = 1.078 \frac{V}{mol}$$

Table S7. Summary table for magnetite suspensions with and without added aqueous Fe(II).

Experiment	*n*	pH	Fe(II) initial (μM)	Clean Fe(II) initial[a] (μM)	Fe(II) final (μM)	ΔFe(II)[b] (μM)	Ionic Strength	Activity[c]	$E_{measured}$ (mV vs. SHE)	Standard Deviation $E_{measured}$ (mV vs. SHE)
Mag alone	*3*	5.6	14	0	964	964	0.0279	0.554	85	4.3
	3	6.2	21		715	715	0.0271	0.557	-26	1.1
	3	6.5	47		730	730	0.0272	0.557	-49	1.5
	3	7.0	27		391	391	0.0262	0.562	-176	7.3
	3	7.5	4		166	166	0.0255	0.565	-234	4.1
	3	8.0	26		79	79	0.0252	0.566	-307	1.5
	3	8.5	2		2	2	0.0250	0.567	-328	2.1
	3	9.1	18		21	21	0.0251	0.567	-408	5.2
	3	9.5	28		2	2	0.0250	0.567	-400	1.0
	3	10.0	34		28	28	0.0250	0.567	-414	17.1
Mag 0.2 mM	3	6.5	558	181.3 ± 8.2	1094	913	0.0283	0.552	-103	13.4
	3	7.1	254		592	411	0.0268	0.559	-184	5.5
	3	7.6	384		403	222	0.0262	0.561	-278	5.4
	3	8.0	100		159	-22	0.0255	0.565	-318	3.0
	3	8.4	155		33	-148	0.0251	0.567	-375	1.3
Mag 1 mM	3	6.5	1081	868.2 ± 115.1	1623	755	0.0299	0.545	-87	4.3
	3	7.1	986		1265	397	0.0288	0.550	-194	8.0
	3	7.6	1003		893	25	0.0277	0.555	-292	7.8
	3	8.0	1018		657	-211	0.0270	0.558	-349	11
	3	8.4	853		541	-327	0.0266	0.559	-437	3.9
Mag 2.5 mM	3	6.5	error	2243.1 ± 177.5	2576	333	0.0327	0.534	-95	7.6
	3	7.1	2388		2257	14	0.0318	0.538	-215	3.6
	3	7.6	2219		2108	-135	0.0313	0.539	-316	2.9
	3	8.0	1962		1832	-411	0.0305	0.543	-389	2.7
	3	8.5	2038		1692	-551	0.0301	0.544	-478	9.4
Mag 5 mM	3	6.5	4042	4598.6 ± 117.5	4854	256	0.0396	0.511	-129	10.5
	3	7.0	4542		4676	78	0.0390	0.512	-231	5.9
	3	7.5	4459		4293	-305	0.0379	0.516	-339	11.1
	3	8.0	4459		4130	-469	0.0374	0.518	-405	2.4
	3	8.4	4368		3847	-752	0.0365	0.520	-541	7.4
Mag 10 mM	3	6.5	9003		9496	461	0.0535	0.474	-139	7.6

3	7.0	9077		9104	70	0.0523	0.476	-245	8.2
3	7.5	9690	9034.5	9399	-682	0.0501	0.482	-352	8.1
3	7.9	8155	± 24.2	8624	-411	0.0509	0.480	-422	2.6
3	8.3	8752		7910	-1124	0.0487	0.485	-587	9.5

a. Clean Fe(II) initial data was collected from buffer and aqueous Fe(II) alone with no magnetite present.

b. ΔFe(II) was calculated as Fe(II) final – Clean Fe(II) initial

c. Activity was calculated for each condition with the extended Debye-Huckel equation.[12]

References:

1. OECD; Agency, N. E., *Chemical Thermodynamics of Iron, Volume 13/1*. 2014.
2. Navrotsky, A.; Mazeina, L.; Majzlan, J., Size-Driven Structural and Thermodynamic Complexity in Iron Oxides. *Science* **2008,** *319* (5870), 1635-1638.
3. Rickard, D.; Luther, G. W., Chemistry of Iron Sulfides. *Chemical Reviews* **2007,** *107* (2), 514-562.
4. Langmuir, D., *Aqueous Environmental Geochemistry*. Prentice Hall: 1997.
5. Stumm, W.; Morgan, J. J., *Aquatic Chemistry: Chemical Equilibria and Rates in Natural Waters*. Wiley: 1996.
6. Robie, R. A.; Hemingway, B. S. *Thermodynamic properties of minerals and related substances at 298.15 K and 1 bar (10^5 pascals) pressure and at higher temperatures*; 2131; 1995.
7. Stewart, S. M.; Hofstetter, T. B.; Joshi, P.; Gorski, C. A., Linking Thermodynamics to Pollutant Reduction Kinetics by Fe(II) Bound to Iron Oxides. *Environmental Science & Technology* **2018,** *52* (10), 5600-5609.
8. Gorski, C. A.; Edwards, R.; Sander, M.; Hofstetter, T. B.; Stewart, S. M., Thermodynamic Characterization of Iron Oxide–Aqueous Fe(II) Redox Couples. *Environmental Science & Technology* **2016,** *50* (16), 8538-8547.
9. Gorski, C. A.; Edwards, R.; Sander, M.; Hofstetter, T. B.; Stewart, S. M., Thermodynamic Characterization of Iron Oxide–Aqueous Fe2+ Redox Couples. *Environmental Science & Technology* **2016,** *50* (16), 8538-8547.
10. Joshi, P.; Fantle, M. S.; Larese-Casanova, P.; Gorski, C. A., Susceptibility of Goethite to Fe(II)-Catalyzed Recrystallization over Time. *Environmental Science & Technology* **2017,** *51* (20), 11681-11691.
11. Roden, E. E., Geochemical and microbiological controls on dissimilatory iron reduction. *Comptes Rendus Geoscience* **2006,** *338* (6), 456-467.
12. Brezonik, P. L., & Arnold, W. A., *Water Chemistry: An introduction to the chemistry of natural and engineered aquatic systems*. Oxford University Press: 2011.
13. Jungcharoen, P.; Pédrot, M.; Heberling, F.; Hanna, K.; Choueikani, F.; Catrouillet, C.; Dia, A.; Marsac, R., Prediction of nanomagnetite stoichiometry (Fe(II)/Fe(III)) under contrasting pH and redox conditions. *Environmental Science: Nano* **2022**.
14. Culpepper, J. D.; Scherer, Michelle M.; Robinson, T. C.; Neumann, A.; Cwiertny, D.; Latta, D. E., Reduction of PCE and TCE by magnetite revisited. *Environmental Science: Processes & Impacts* **2018,** *20* (10), 1340-1349.
